# Measuring sustainability of seed-funded earth science informatics projects

**DOI:** 10.1371/journal.pone.0222807

**Published:** 2019-10-23

**Authors:** Leslie Hsu, Vivian B. Hutchison, Madison L. Langseth

**Affiliations:** U.S. Geological Survey, Science Analytics and Synthesis, Denver, Colorado, United States of America; King Abdulaziz University, SAUDI ARABIA

## Abstract

Short term funding is a common funding model for informatics projects. Funders are interested in maximizing the sustainability and accessibility of the outputs, but there are no commonly accepted practices to do so in the Earth sciences informatics field. We constructed and applied a framework for sustainability drawing from other disciplines that have more published work focusing on sustainability of projects. This framework had seven sustainability influences (outputs modified, code repository used, champion present, workforce stability, support from other organizations, collaboration/partnership, and integration with policy), and three ways of defining sustainability (at the individual-, organization-, and community-level). Using this framework, we evaluated outputs of projects funded by the U.S. Geological Survey’s Community for Data Integration (CDI). We found that the various outputs are widely accessible, but not necessarily sustained or maintained. Projects with most sustainability influences often became institutionalized and met required needs of the community. Even if proposed outputs were not delivered or sustained, knowledge of lessons learned could be spread to build community capacity in a topic, which is another type of sustainability. We conclude by summarizing lessons for individuals applying for short-term funding, and for organizations managing programs that provide such funding, for maximizing sustainability of project outcomes.

## Introduction

Short-term seed funding, designed to get a project idea off the ground in 6 to 24 months, is a common funding model for realizing informatics and data-related benefits for scientific communities (Table 1). Projects that attain seed funding must demonstrate useful outcomes in a short time frame if they wish to secure further sources of funding for sustainment, such as server costs, staff to keep up with security requirements, user support, and software updates. In the current scientific research landscape, technology and tools to support the collection, analysis, and display of scientific data are an essential component of the scientific process, and seed funds are often used to support development, but not maintenance, of these tools for scientific research [1]. Projects in this area of tool development or cyberinfrastructure, where the product must exceed proof of concept and be operationalized to reach its full potential, face different sustainability needs than those of traditional scientific studies that end with a journal publication.

**Table 1:** Examples of short-term seed funding opportunities that are available for Earth science informatics projects.

Organization	Short-term Funding Opportunity Information
U.S. Geological Survey Community for Data Integration	https://my.usgs.gov/confluence/display/cdi/Proposals
Earth Science Information Partners Lab	https://www.esipfed.org/esip-lab
National Science Foundation EarthCube	https://www.nsf.gov/geo/earthcube/
National Science Foundation EAGER (EArly-concept Grants for Exploratory Research)	https://www.nsf.gov/pubs/policydocs/pappguide/nsf15001/gpg_2.jsp#IID2
Alfred P. Sloan Foundation	https://sloan.org/grants/apply

Some examples of these non-traditional products include demonstration and documentation of a new open source framework for visualization [2], a web system allowing users to easily compose and execute full-stack deep learning workflows [3], and a knowledge base for guidance on best practices for data collection and management for a particular geoscience discipline [4]. Recently, community discussion about the impact, success, and sustainability of these informatics products has become more common. For example, the EarthCube Annual Meeting had sessions in 2018 (“What is success?”) and 2019 (“How should EarthCube think about metrics of success”) to address the issue of project and product sustainability.

Seed funding is an attractive model because it allows funders to fund several small experiments at low investment, so funders do not suffer a large loss if some of the projects do not deliver their proposed outcomes. There is potential for high return on a few successful projects. However, seed funding has led to many short-term, finite-length projects that lacked additional funding to improve upon and promote their products to the point of acceptance and use by its intended community [5]. Funders usually want to minimize this situation and maximize the success and sustainability of the projects that they select. Additionally, in the case of federal funding, high success rate is critical for demonstrating accountability to taxpayers.

Here, we analyze seven years of projects funded by the U.S. Geological Survey’s Community for Data Integration (CDI), determining factors that contributed to the sustainability of project outputs. As far as we are aware, this is the first application of this type of framework to evaluate an Earth Science informatics seed funding program. Many of the funding initiatives in Table 1 have been in existence for approximately 5–10 years and the opportunity now exists to quantitatively analyze the sustainability of their outputs.

The CDI is a community of practice that helps its members grow expertise on all aspects of working with scientific data [6]. The community is coordinated by the U.S. Geological Survey (USGS), with the goal of increasing the capacity for USGS data integration and management. CDI’s activities help support the USGS mission to “provide reliable scientific information to describe and understand the Earth; minimize loss of life and property from natural disasters; manage water, biological, energy, and mineral resources; and enhance and protect our quality of life.” The CDI has approximately 1000 members, including data managers, information technology professionals, research scientists, data scientists, software developers, managers, and students. The CDI’s main activities are volunteer-led collaboration areas, workshops, trainings, and an annual project proposal process.

Since 2010, the CDI has funded projects focusing on data integration for interdisciplinary research, innovative data management, and demonstration of new technology within the USGS’s computing ecosystem. Between 2010 and 2016, the CDI funded 60 projects (S1 Table). Projects funded more recently (2017 to present) are excluded from this study because the time elapsed is shorter than needed for our goals in evaluating sustainability.

As facilitators and advocates of the CDI, we desire to help the CDI funded projects achieve maximum use and sustainability. Sustainability is a major element of the perceived success of funded projects and tools, thus frameworks and analyses of sustainability have been researched in disciplinary science fields. Two fields that have visible literature on sustainability of programs and products are health science [7, 8, 9] and scientific software and cyberinfrastructure [1, 10, 11, 12]. These precedents provide conceptual frameworks that we modify in this study for our geoscience informatics focus.

### Lessons from the health science field

Several frameworks for analyzing sustainability originate from the health sciences field—examining practices, programs, and interventions [7, 8, 9]. Though different in subject matter, the health sciences program analysis is relevant to CDI projects because it evaluates resources designed to improve patient health, whereas CDI products are designed to improve user or practitioner data integration and management ability. Both funding types are often designed as seed-funded or demonstration programs. The discussion of the definition, reasons for, influences on, and elements of sustainability in community-based health programs closely parallels that for CDI products [7]. Shediac-Rizkallah and Bone [7] identify three conceptual measures for sustainability—at the individual, organization, and community level—which we slightly modify for our analysis here. Schreier [8] analyzed 19 empirical health studies and examined 11 factors reported to be related to the extent of program sustainability. They found consistent support for five important factors influencing the extent of sustainability: (a) a program can be modified over time, (b) a “champion” is present, (c) a program “fits” with its organization’s mission and procedures, (d) benefits to staff members and/or clients are readily perceived, and (e) stakeholders in other organizations provide support. Stirman et al. [9] reviewed 125 studies on sustainability of healthcare programs and identified 24 influences on sustainability. Among their most frequently identified influences were: (1) leadership, (2) workforce (staffing, attributes), (3) collaboration/partnership, and (4) ongoing support.

### Lessons from the scientific software and cyberinfrastructure field

Another framework for sustainability comes from the field of scientific software and cyberinfrastructure [1, 10, 11, 12]. These studies are motivated by the realization that the scientific research community suffers when software is not developed and maintained in a sustainable way [1, 5, 10, 11, 12]. In this context, sustainability is the need to create software that lives beyond the project period by which its creation is funded. Some CDI products include software and cyberinfrastructure, therefore the considerations and recommendations to funders in these studies are directly applicable.

Wernert et al. [11] administered a survey aimed to identify best practices and models required for developing, deploying, and supporting robust, sustainable cyberinfrastructure software, and to identify key factors users consider in software adoption. They found that the most often cited factors required for a software product to be considered sustainable were (1) compatibility, (2) availability of support resources, and (3) an active development process. These factors contrasted with the factors more important for software adoption, which were (1) capabilities, (2) total cost of ownership, (3) long-term availability, (4) reliability/maturity, and (5) initial purchase cost.

Stewart et al. [10] summarizes the activities, discussion, and consensus of a two-day workshop on Cyberinfrastructure Software Sustainability and Reusability. They report 12 findings and 14 recommendations to the U.S. National Science Foundation on the topic, and lay out characteristics of software development teams and processes necessary to create sustainability. The characteristics include points to consider for planning, software development methodology, and promoting adoption of the software. We draw from the existing models of sustainability and metrics of use discussed by Stewart et al. [10] to evaluate software products generated by CDI-funded projects.

### Defining sustainability for Earth science informatics projects

Products from CDI funded projects include technical tools, best practice methods, and data products, overlapping similar outputs produced in health sciences and software studies. We take the multi-level definition of sustainability for community health programs defined in Shediac-Rizkallah and Bone [7] and modify it for our use case. For our study, project sustainability is evaluated at the individual level, organization level, and community level. Individual-level sustainability means that benefits to the user continue after project funding ends. Organization-level sustainability means institutionalization or adoption of a tool or best practice within an organization after project funding ends. Community-level sustainability means that there has been an increase in the capacity of the Earth and biological science community to work with data for scientific research through adoption of the technologies or knowledge gained (Table 2).

**Table 2:** Categories of sustainability evaluated in this study (following Shediac-Rizkallah and Bone [7]).

Type	Description	Example
individual-level sustainability	benefits to the user continue after project funding ends	A web application is maintained online by the original project team; a new data collection is issued a persistent identifier (such as a digital object identifier (DOI)) and resides in a trusted digital repository
organization-level sustainability	institutionalization within an organization	An organization within the USGS brings a software product into the suite of maintained tools
community-level sustainability	capacity building of a community	A well-cited and frequently updated website provides methods for meeting data policy, and examples and best practices in data management for the Earth and biological science disciplines.

Lead principal investigators of the CDI proposals are often trained as geoscientists, not as software, information management, or cyberinfrastructure professionals. Though the proposals may include team members that are more technically trained, projects often do not have senior personnel with much experience building and maintaining technical products. Recognizing this potential deficiency and in the attempt to promote sustainability of informatics products, the aim of this work is to show examples of successes from seed funding, examine exceptions, highlight trends, and explain what factors lead to greater use and sustainment of the project outputs. This analysis yields lessons for those applying for short term grants and guidance for managing programs that provide informatics-related seed-funding for scientific communities.

## Data and methods

### Our dataset for evaluating sustainability: The Community for data integration funded projects

The CDI annual proposals process supports collaborative projects that follow its guiding principles:

- Focus on targeted efforts that yield near-term benefits to Earth and biological science
- Leverage existing capabilities and data
- Implement and demonstrate innovative solutions (e.g., methodologies, tools, or integration concepts) that could be used or replicated by others at scales from project to enterprise
- Preserve, expose, and improve access to Earth and biological science data, models, and other outputs
- Develop, organize, and share knowledge and best practices in data integration.

Details of the proposal process have changed from year to year, but the following features have been consistent for several years. Proposals may request up to $50,000 for work proposed to be carried out during one fiscal year (October 1 to September 30). The budget must include at least 30% of requested funds as in-kind contributions (such as salary or funding from other grants) to demonstrate the project team’s commitment to the project. In one year (2014), three projects were awarded a second year of funding to complete their deliverables. However, the projects are usually funded strictly for a single year, and any related follow-on projects must be submitted as a new proposal. That is, the CDI funds are not intended for sustainment of previously developed CDI outputs.

From 2010 to 2012, projects were evaluated, selected, and approved by the CDI sponsors, who are members of the USGS Executive Leadership Team. In 2013, proposals were evaluated by an internal USGS review panel. Starting in 2014, projects have been selected in a two-phase process. In phase 1, short statements of interest submitted by applicants can be commented and voted on by any member of the CDI. In phase 2, the highest-ranked statements from community voting produce a full-length proposal that is evaluated by a formal internal USGS review panel. The proposals process was expanded from a CDI members-only process to a USGS-wide call for proposals in 2013. Although the lead principal investigator for each proposal must be a USGS employee, other project personnel may be external–from other government agencies, academia, or non-profit or commercial organizations. From 2013–2016, 179 submissions were received and the average annual funding rate was 24%. Table 3 shows the number of funded projects and total funds awarded from 2010 to 2016.

**Table 3:** Number of funded projects and total funds awarded by the CDI from 2010 to 2016.

Year	Number of Projects	Total Funds Awarded ($K)
2010	4	500
2011	6	228
2012	10	479
2013	10	430
2014	9	437
2015	8	463
2016	13	574

Types of outputs from the CDI projects vary widely because there are many ways to accomplish the CDI goals of knowledge transfer and increased capabilities in data integration. For this study, we examined CDI project outputs proposed from 2010–2016 and categorized them into nine groups: data release, mobile application, presentation, publication, software, source code, web application, web link, and web service, as defined in Table 4. We distinguish between these types of outputs because (1) users browsing CDI outputs may be looking for a certain format, such as a publication or source code, (2) the sustainability requirements of the output types are different, such as the need for a software developer or for server costs, and (3) the requirements for implementing different output types in institutional infrastructure may be quite different, such as security requirements for a mobile application versus a web service. Examples for each of these types are highlighted later in the paper and in the supporting S1 File.

**Table 4:** Community for data integration project output types and definitions.

Output Type	Definition
Data Release	USGS scientific data released to the public
Mobile Application	Interactive application built specifically for a mobile device
Presentation	Slides, video, or other presentation media
Publication	Peer-reviewed publication (USGS or external journal publication)
Software	Executable or compiled code that can be downloaded and run locally
Source Code	The project's source code, made accessible in a public code repository
Web Application	Interactive application that runs on a web browser
Web Link	Project webpage, wiki page, white paper published on the web, or other online resources that do not fit into another category
Web Service	A service endpoint URL that may be accessed by a client application

The CDI has promoted the discussion of sustainability in its proposal guidance to applicants. Each proposal is evaluated for funding based on six criteria: scope; technical approach; project experience and collaboration; sustainability, outreach, and communication; budget justification; and timeline, as stated in the proposal guidance. Fifteen-percent of the evaluation score is based on how well the proposal describes the intended sustainability, outreach, and communication of the project deliverables for long-term access, reusability, and potential for integration with other data and in different systems. The various output types (Table 4) have different metrics and considerations for sustainability. For example, data and documents can usually be preserved indefinitely by publishing them with a persistent identifier (PID, such as a Digital Object Identifier, DOI) in a trusted digital repository [13, 14]. CDI teams have been able to use the USGS DOI Tool (https://www1.usgs.gov/csas/doi) and designated trusted digital repository (https://www.sciencebase.gov) since 2015, which is in the middle of our study period. For tools, the source code can be published and archived, but the executable file may not run unless it is in the proper environment. For some funded outputs and products, a measure of sustainability of a tangible object may not be applicable; instead, knowledge transfer and capacity building of the community for a specific skill or topic is more relevant, though harder to measure.

For this study, we designed a framework of relevant sustainability influences, drawing from the previous work in the health science and scientific software and cyberinfrastructure fields. An extensive list of influences on sustainability have been identified by previous studies [7, 8, 9, 10]. Out of this list, we selected seven influences based on their relevance for the types of outputs that CDI projects produce, and our ability to evaluate the influence (Table 5). These influences are: (1) outputs modified, (2) code repository used, (3) champion present, (4) workforce stability, (5) support from other organizations, (6) collaboration/partnership, and (7) integration with policy. Many of the seven influences have been noted as important for sustainability by multiple studies. In the framework of Schreier [4], these influences fall into three categories: project design, organizational setting, and community environment (Table 5). We hypothesize that specific factors, such as a larger number of collaborating organizations, lead to greater sustainability of outputs in our community’s projects. The full list of sustainability influences from references 7–10 and our reasons for including or excluding them in this study are explained in S2 Table.

**Table 5:** Influences on sustainability, with their category, the definitions used in this study, and references to similar influences from other studies.

Influence on sustainability	Category	Definition for this study	References
**Outputs modified**	Project design	Project outcome has been modified or evolved to meet slightly different or new needs by other groups. Outcome is built upon by a different group.	[8, 9]
**Code repository used**	Project design	Code generated by the project is placed in an accessible (public) version-controlled repository.	[10]
**Champion present**	Organizational setting	There is a known project champion that has advocated for the use of the project in future initiatives.	[7, 8, 9]
**Workforce stability**	Organizational setting	Principal investigator and essential staff remain in a position to support the project through its duration.	[9, 10]
**Support from other organizations**	Community environment	The project has obtained support from other organizations beyond the CDI-funded proposal in the form of in-kind or dedicated resources, expert advice, or promotion to users. Does not include the required in-kind funding of the project proposal.	[8, 9]
**Collaboration/Partnership**	Community environment	Multiple science centers, organizational units, or partners are involved on the budget.	[8, 9]
**Integration with Policy**	Community environment	Project outputs help implement policy.	[7, 8, 9]

There are many previously identified influences that we do not consider in this study, for a number of reasons. Some influences were not used because they are standard across all CDI projects, such as funding type and project funding duration. Some influences were not used because of the difficulty to evaluate them, that is, there is no documentation of those influences for all projects. These include presence of training, socioeconomic and political considerations, community participation, shared decision making with stakeholders, and evaluation and feedback. Finally, some influences were not used because the influence is embedded in the evaluation criteria for project selection, and all funded projects are expected to have these influences. These influences include positive evaluation, fit with mission tasks, perceived benefits to staff, and project financing.

Data were compiled for each CDI funded project from 2010 to 2016. The data compiled included project title, current point of contact, organizational unit of project personnel, proposed outputs and type, delivered outputs and type, and current accessibility of the outputs. First, the authors searched for this information online and within the CDI wiki space (https://my.usgs.gov/confluence/display/cdi/Home), which serves as the CDI record-keeping mechanism. Next, all project principal investigators were contacted via email asking for any further project outputs and their observations on accessibility and sustainability of their project outcomes (S1 Text). The information requested included the current point of contact, if the project outputs are still accessible or operational, if funding from organizations besides CDI have been used, and if the principal investigators had other comments about the impact and sustainability of their project. We followed up one time, but did not pursue the inquiry if the contact did not answer after two communication attempts. Our response rate was 88%. For the remaining 12%, only the information discovered online and within the CDI wiki space was used.

We analyzed the CDI projects for the presence or absence of the seven sustainability influences. Each project was assigned a “yes” or “no” for each sustainability influence in Table 5, then for each influence, the percentage of projects with a “yes” value was reported. We searched for trends in which influences were most present, and determined how this was related to the self-reported and researched accessibility and sustainability of project outcomes. The three authors, who have each participated in the CDI in different roles and time periods, individually determined the presence of sustainability influences and types, then consolidated their results.

We also examined how the make up of collaborations and the project output types were related to different influences on sustainability and types of sustainability. We report on representative projects that exemplify the different influences on and types of sustainability.

## Results

### Summary of outputs from the CDI projects

**Fig 1:**
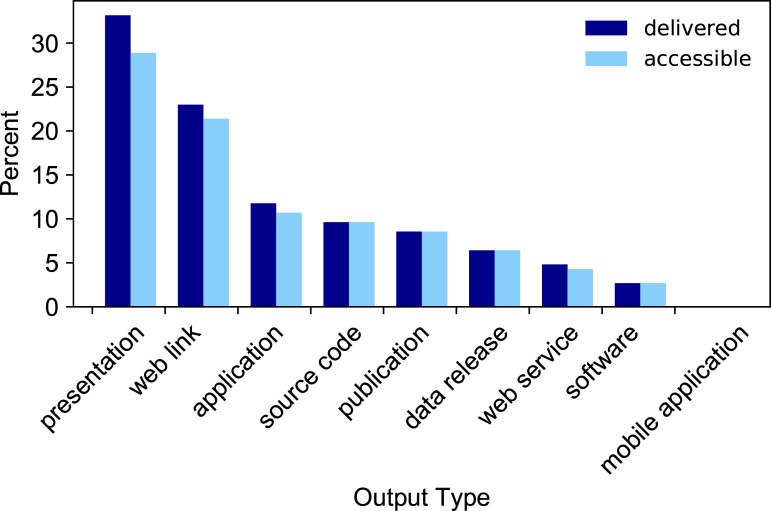
Percentage and type of CDI project outputs generated from 2010–2016. Values shown are output types delivered (dark blue) and output types that remain accessible at the time of this study (light blue), expressed as a percentage of the 187 delivered products.

We found 187 deliverables reported or discovered online resulting from the 60 CDI projects (**Fig 1, [15]**). Of these, the most populous output type was presentation (62), likely because CDI requires projects to give a presentation to the CDI on their outcomes, and the CDI facilitators document those slides and recordings on the CDI wiki. The next largest output type is web link (43), which includes webpages, wiki pages, white papers, or other online resources that do not fit into another category. Out of the 187 links, 16, or 9%, are no longer accessible to users. Of the 16 links that are no longer accessible, 8 are presentations that were not collected and placed on the wiki, 3 are web links, 3 are web applications, 1 is source code from a repository URL that no longer resolves, and 1 is a web service that is no longer available.

**Fig 2:**
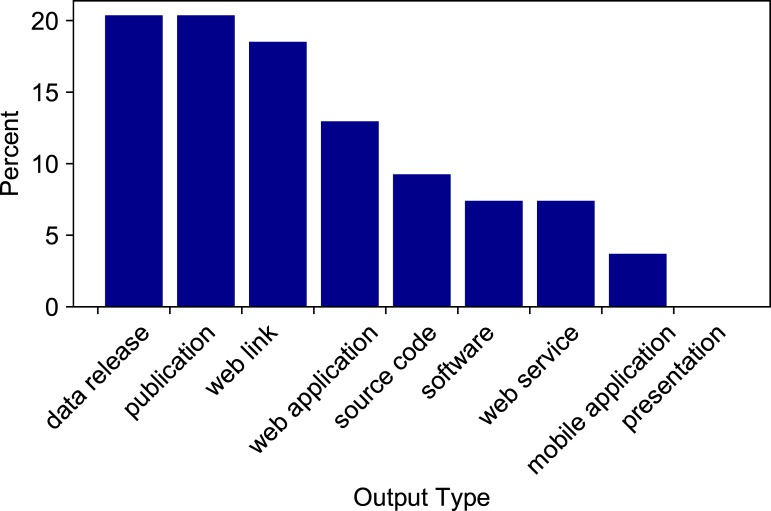
Percentage of CDI Project outputs proposed but not delivered, by type. Values shown are output types, expressed as a percentage of the 54 outputs that were proposed but not delivered.

Some deliverables proposed at the outset of the project were not completed, and we evaluated these output types. The output types that were most commonly not delivered were peer-reviewed publications and data releases (11 each), web links (10), web applications (7), and source code (5) (**Fig 2**). Thirty-two out of 60 projects delivered all proposed outputs. See accompanying data [15] for further detail. The project outputs are documented on USGS ScienceBase, a trusted digital repository, which serves as the official and current information source for CDI projects (https://www.sciencebase.gov/catalog/item/520e8340e4b08494c3cb34ec).

### Prevalence of sustainability influences

**Fig 3:**
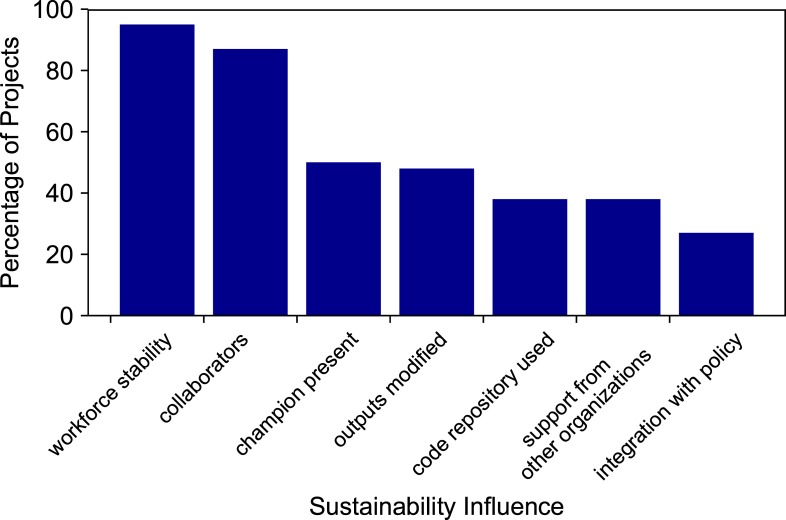
Percentage of projects possessing each sustainability influence.

**Fig 4:**
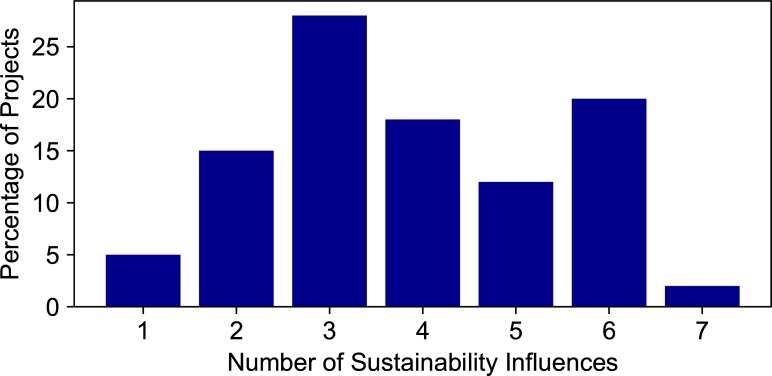
Percentage of projects with a certain number of sustainability influences.

Our evaluation determined the prevalence of each sustainability influence in the CDI project dataset (Fig 3). The positive sustainability influence observed in the most projects was workforce stability (97%). Because the duration of the project is less than one year, it is common for teams to maintain workforce stability. We note that there are instances when essential project personnel have left their positions in the USGS after the project duration, and the related digital tools or applications lost their sustainability and maintenance. Another widespread influence was the presence of collaborators or partners outside of the USGS science center (an organizational unit within the USGS with common administrative resources) securing CDI project funding, at 87%. The presence of partners and collaborators is encouraged in the proposal guidance and leads to more favorable review. Fifty percent of the projects had an identified champion who continued to advocate for project outputs to be integrated into current and new project opportunities. Forty-seven percent of the projects had their outputs modified or built upon by a subsequent project. This high percentage could exist because proposals are rated favorably according to the review criteria if they build upon existing resources. Thirty-eight percent of projects used a public code repository on a platform such as GitHub, GitLab, or Bitbucket; more projects are doing so in the recent years as more researchers are becoming familiar with repository options for code. Thirty-seven percent of projects had monetary support from organizations other than the CDI during or after the project, in addition to the in-kind funds required for the project. Twenty-seven percent of projects had their output in some way integrated into USGS data policy. This statistic reflects the membership of the CDI, which has many members that are enthusiastic about exposing USGS data and science, meeting federal open data directives, and satisfying new data policy requirements. Most projects had between two and six of the sustainability influences (Fig 4). A little over 20% of projects reached six or seven influences, and three projects had only one of the influences.

### Individual, organization, and community-level sustainability

#### Individual-level sustainability

Up to now, we have reported on accessibility of outputs, but accessibility and sustainability are not the same. The definition of individual-level sustainability includes the phrase “maintenance of benefits.” To illustrate this, although 97% of the projects have at least one accessible link to an output (only two do not), this does not necessarily mean the project has achieved individual-level sustainability. The links counted include oral presentations given to the CDI community, which are a requirement of CDI project funding and the digital presentation files are maintained and made publicly accessible by the CDI staff. Therefore, in determining individual-level sustainability, we did not consider only the accessibility of an output link, such as the project presentation. Instead, we evaluated if the original intended capability of the project was delivered, and if that benefit has been kept up to date with current operating systems, security requirements, and software dependencies. This results in 46 projects, or 77% reaching the level of individual sustainability (Table 6).

**Table 6:** Number and percentage of projects with individual-level sustainability or organizational-level sustainability at the time of this study.

Type of sustainability	Number of projects	Percentage of projects
individual-level sustainability	46	77
organization-level sustainability	25	42
community-level sustainability	see text	see text

One example is the USGS National Water Information System (NWIS) Snapshot tool, funded in years 2010–2012 [16]. The tool worked in the Esri ArcGIS environment. ArcGIS produces new software versions every few years, and the snapshot tool needed to be updated periodically to remain compatible with the new versions. The project principal investigator left the USGS, and without a project champion, funding allocated by the USGS science center was cut. Without funding to keep this tool up to date, it stopped being updated after version 2.1, and now is no longer easily usable, though the old code still is accessible in a public code repository.

A contrasting example is the Metadata Wizard [17], which also had a dependency on ArcGIS. However, there was funding to keep the application up to date with new versions, and it was kept current for five years until a new stand-alone version of the tool was developed with independent funding.

#### Organization-level sustainability

Organization-level sustainability was met by 25 projects (42%), meaning that a USGS program, other than the CDI, supplies continued funding or staff support to keep the project output(s) online and up to date. In the USGS, certain programs have an operational or science support focus, as opposed to a research focus; these programs include the National Geospatial Technical Operations Center and the Science Analytics and Synthesis (SAS) Science Data Management Branch. These are examples of programs that have sustained some CDI project outputs. Other project outputs deliver information to stakeholders, for example for phenological [18] and earthquake hazards [19] data, and have been maintained by programs that require that ability. Another example of a project output that has reached organization-level sustainability is an open source data visualization tool used by various USGS multi-disciplinary efforts that has been configured for USGS application requirements [2].

One major reason why some outputs are maintained at the organization level is that they help to meet a specific policy or requirement for USGS researchers, for example requirements defined in the Bureau’s Public Access Plan [20]. As the plan and its accompanying policies were implemented, tools such as the Data Management Website [21], the Science Data Lifecycle [22], and the Metadata Wizard [17], that helped scientists to meet the USGS data release and documentation requirements, were supported and built upon by the SAS Science Data Management Branch. The Data Management website and the Science Data Lifecycle have also been incorporated into the policies of the USGS Fundamental Science Practices, which outlines a set of practices, philosophical premises, and operational principles as the foundation for all USGS research and monitoring activities [23].

#### Community-level sustainability

Community-level sustainability (capacity building of a community) is difficult to measure in a standardized way, so we do not quantify it here. However, capacity building for integrating and managing Earth science and biological data for the CDI and USGS is one of the criteria for evaluation of the CDI proposals, so there are many examples of projects with outputs that benefit a significant portion of the USGS research community.

Some examples of broad, far-reaching CDI efforts include a well-documented initiative and prioritization scheme for rescuing legacy data [24], recommendations for meeting new USGS data policy [25], and the USGS Data Management Website, an informational website that is meant to help researchers learn resources for data management [21]. It is possible to have community-level sustainability without having individual-level sustainability, meaning that community capacity has been increased, but there is not a concrete deliverable that continues to benefit the originally-intended user. For example, in 2012, a funded CDI project developed a prototype of a developmental web service for integrating data from different USGS sources, but the web service is no longer operational at this time. However, the knowledge gained is shared through a USGS Open File Report, “A case study of data integration for aquatic resources using semantic web technologies” [26].

### What factors lead to sustainability?

Among the CDI projects funded from 2010–2016, we have three defined groups of projects: 1) all projects (60), 2) projects that achieved individual-level sustainability (46), and 3) projects that achieved organizational-level sustainability (25). What are the differences between these three groups? What factors led to a more sustained outcome?

#### Number of sustainability influences

**Fig 5:**
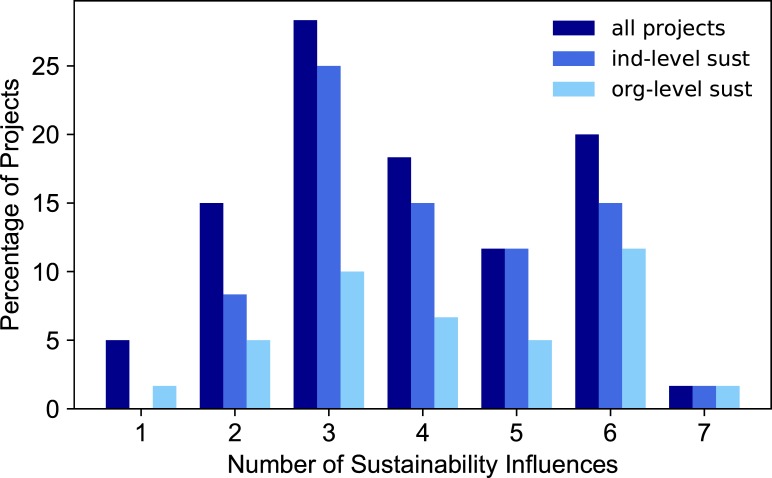
Percentage of projects with a certain number of sustainability influences and sustainability level achieved. The funded CDI projects are grouped in categories 1) all projects (dark blue), 2) projects achieving individual-level sustainability (medium blue), and 3) projects achieving organization-level sustainability (light blue).

Do we see a threshold of number of sustainability influences, above which most projects achieve individual-level or organization-level sustainability? The project with the highest number of sustainability influences, all seven considered in this study, reached both individual-level and organization-level sustainability. Projects with three or more sustainability influences had a higher rate of achieving individual-level sustainability than those with one or two influences. Projects with six or more sustainability influences had a higher rate of achieving organizational-level sustainability than those with fewer influences. However, there is no clear threshold, we do not see that projects possessing more than a particular number of sustainability influences consistently achieve a higher level of sustainability (Fig 5).

#### Specific sustainability influences

**Fig 6:**
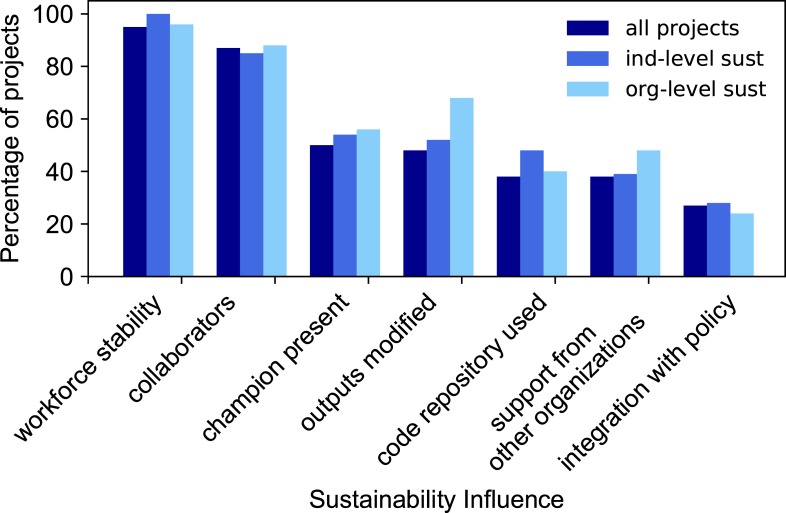
Percentage of projects from each of the three groups with a particular sustainability influence. Dark blue: the group of all projects, medium blue: projects that have achieved individual-level sustainability, light blue: projects that have achieved organization-level sustainability.

Evidence does not indicate that certain sustainability influences were more important for achieving individual-level or organization-level sustainability. In Fig 6, the y-axis shows the percentage of each of the defined groups that had each specific sustainability influence on the x-axis. The percentage is very similar for all three groups for all sustainability influences (the three grouped bars are all within 10 percentage points of each other). The influence with the biggest difference among the three project groups is outputs modified. This influence increases in each subgroup–all projects (48%), individual-level sustainability (52%), and organization-level sustainability (68%).

**Fig 7:**
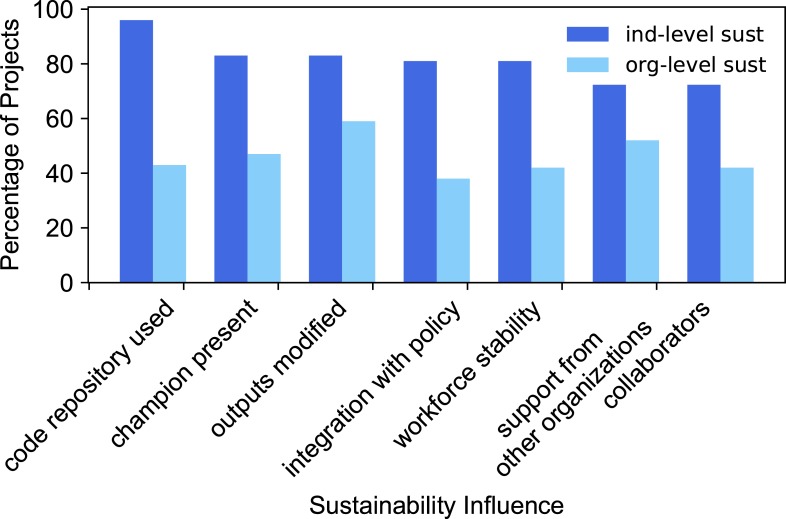
Percentage of projects with specific sustainability influences that reached individual-level and organization-level sustainability. Medium blue: projects with individual-level sustainability, light blue: projects with organization-level sustainability.

To achieve further clarity about differences in the factors related to specific sustainability influences, we compared projects that achieved individual-level and organization-level sustainability. Do specific sustainability influences always map to individual-level or organization-level sustainability? The closest observation of this is that 96% of projects with a public code repository reached individual-level sustainability (Fig 7). The other influences all have values of 75–83% of projects reaching individual-level sustainability. For organization-level sustainability, the influence with the highest percentage is outputs modified: 59% of projects whose outputs were modified by a later project reached organization-level sustainability. The other influences all have values of 38–52% of projects reaching organization-level sustainability.

#### Number of collaborating groups involved in the proposal

Is sustainability more likely if members of different organizational groups collaborate? CDI projects are encouraged to form collaborative teams of people with diverse expertise who would not normally be funded to work together under the USGS funding structure. The CDI encourages communication across organizational boundaries, leading to diversity of disciplinary and technical expertise. This diversity is hoped to strengthen proposals by having a variety of bodies of knowledge and potential in-kind contributions or future funding. We looked at two different types of collaborating groups—the USGS mission areas and external partners.

USGS mission areas are organized around major societal issues that USGS science is poised to address. In the period of our study, from 2010 to 2016, there were seven mission areas: Core Science Systems, Climate and Land Use Change, Energy and Minerals, Environmental Health, Ecosystems, Natural Hazards, and Water. Partners from external organizations include other federal and state government agencies, an Australian government agency, and academic (.edu), non-profit (.org), and commercial (.com) organizations (see Table 4 of the associated data release and explanatory metadata [15]). Data exists to tabulate the affiliation, by USGS mission area or organization external to the USGS, of each person mentioned as a principal investigator, co-investigator, or collaborating partner on proposals from 2013–2016 (40 of the 60 total projects, as shown in Table 3).

**Fig 8:**
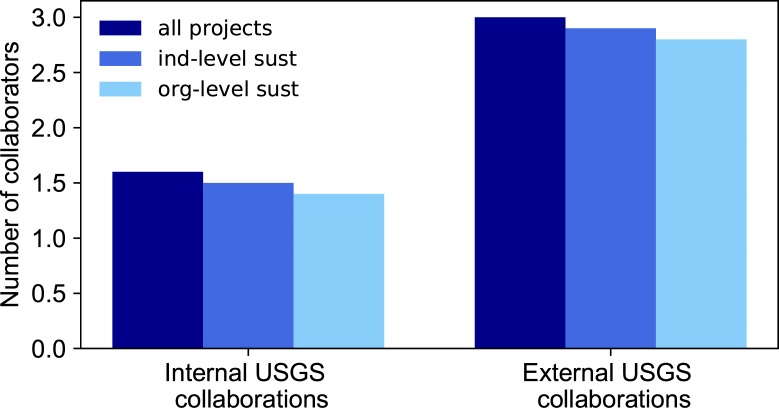
Average number of collaborating groups and level of sustainability. Compared to the pool of all projects (dark blue), projects that have achieved individual (medium blue) or organization-level (light blue) sustainability do not have greater numbers of internal or external collaborations.

We found no notable difference in the average number of mission areas or external partners in our three defined groups (all projects, projects that reached individual-level sustainability, and projects that reached organization-level sustainability) (Fig 8). The number of mission areas represented by collaborators in each of the three groups ranges from 1 to 4 and the average number of mission areas represented is 1.6, 1.5 and 1.4, respectively for the three groups. For external partnerships, the number of collaborators (including the USGS) ranges from 1 to 7 and the average number of collaborators is 3, 2.9, and 2.8 for the three groups, respectively.

#### Success in delivering all products and sustainability

Many organizations fund projects with the intent of sustainability, and also expect delivery of the proposed products. If all proposed products were delivered, are the outputs more likely to be sustained? In our dataset, the delivery of all proposed products does not necessarily lead to sustainment. For all projects, the average percentage of products delivered is 84% (not counting projects that did not deliver any of their proposed products, because then there is nothing to sustain). For both categories of projects achieving individual-level and organization-level sustainability, the average percentage of products delivered is lower, at 73%. We cannot conclude that just because a project delivers everything it promised, there is a higher chance of sustainability.

## Selected examples

We describe examples of CDI-funded projects for three output types: publication, web application, and web link, with their associated products and sustainability influences, to illustrate the range of CDI products and their applications.

### Publication

#### Science data lifecycle

To help preserve and make USGS science accessible, the group developed a Science Data Life Cycle Model that accurately captures how USGS scientists conduct their projects. The model reflects the stages that science data go through from initial selection through collection, preparation, use, dissemination, and final disposition. The group designed a visual representation of the model to articulate its key components, relationships and workflow [22].

Sustainability influences: outputs modified, champion present, support from other organizations, collaborators/partners, integration with policy, workforce stability. This product has since been incorporated into the USGS Fundamental Science Practices [20] that must be followed by all USGS scientists.

Titles of selected citations to this work in the scientific literature:

- The centrality of data: data lifecycle and data pipelines. [27]
- Scientific integrity and ethical considerations for the research data life cycle. [28]
- Smartphone-based distributed data collection enables rapid assessment of shorebird habitat suitability. [29]
- A skeleton data model for geochemical databases at the British Columbia Geological Survey. [30]

### Web application

#### mdtools: Portable International Organization for Standardization (ISO) 19115–2 open source developer's toolkit

mdTools is an interface for Alaska Data Integration Working Group (ADIwg) metadata tools. The purpose of these tools is to assist with the production of scientific metadata, especially ISO-compliant metadata.

https://mdtools.adiwg.org/

Sustainability influences: outputs modified, code repository used, champion present, support from other organizations, collaborators/partners, integration with policy, workforce stability. This tool has become one of the only open tools available for researchers to author comprehensive ISO-compliant metadata, and has been incorporated into plans for transitioning the USGS to ISO-compliant metadata. Additional funding beyond the seed-funding period was critical to bring the tool to a user-ready product.

### Web link

#### Terriajs: Evaluating a new open-source, standards-based framework for web portal development in the geosciences

The project team assessed the role of TerriaJS in the USGS suite of available tools for creating web services. The team made critical enhancements for dealing with meteorologic, oceanographic, and hydrologic model data; tested deployment in the USGS computational environment; and developed several demonstrations using TerriaJS, which allowed them to assess performance and ease of installation and use [2].

Sustainability influences: outputs modified, code repository used, champion present, support from other organizations, collaborators/partners, workforce stability. In addition to supporting existing teams that were interested in this visualization framework, the documented lessons on the project’s public page convinced future CDI projects to evaluate and use the TerriaJS technology for their applications. This has built a larger community of users, pool of expertise, and collection of code and add-ons for TerriaJS users in the USGS.

## Discussion

In this study, we used a newly devised framework of sustainability influences to evaluate the outputs from CDI seed-funded projects. From this evaluation we quantified the sustainability influences that are most prevalent, and which influences are associated with sustained outputs. We found that strict accessibility of an individual output is not the only measure of sustainability, because maintenance of a technical product to current standards and knowledge transfer to the wider community are requirements for continued use and application. This analysis of the CDI projects is one example of using this framework; lessons here will translate to other seed-funded projects in the scientific data and informatics community, with the caveat that each organization’s requirements and constraints will modify some of these findings.

Mapping the sustainability influences examined here to the categories of the CDI proposal evaluation criteria (https://my.usgs.gov/confluence/display/cdi/Proposals), we see that four of the influences fall within Project Experience and Collaboration (Champion present, Workforce stability, Support from other organizations, and Collaborators/Partners), two influences fall under Sustainability, Outreach, and Communication (Outputs modified, Code repository used), and one under Scope (Integration with Policy) (Table 7). These three categories make up 65% of the evaluation criteria, a significant portion. When applying the results found here to other proposal processes, the extent to which the sustainability influences are included into evaluation and scoring would be one differentiating factor between CDI projects and another group of projects.

**Table 7:** Comparison chart of CDI proposal evaluation criteria and sustainability influences.

% of Total Score	CDI Evaluation Criteria	Related Sustainability Influences
25	Scope	Integration with policy
25	Technical Approach	
25	Project Experience and Collaboration	Champion present, Workforce stability, Support from other organizations, Collaborators/Partners
15	Sustainability, Outreach, and Communication	Outputs modified, Code repository used
5	Budget Justification	
5	Timeline	

### Accessibility and sustainability

As we mentioned earlier, delivered web links and their current accessibility does not assure that the original intent of the project was reached. An example is that a version of the deliverable is accessible, but it is out-of-date and not compatible with current operating systems and other dependencies. These cases are rare, but exist in our dataset. Our study covers seven years, whereas operating system or software versions are often updated on a 2–3 year cycle [31, 32].

Three of 43 web links delivered by CDI projects are no longer accessible at the time of this writing. URLs for different organizations within the USGS change as programs evolve through time; but this has not broken a significant number of web links to CDI funded projects at this point. However, the USGS is currently migrating its web content on https://www.usgs.gov to a new content management system, which will likely break many web links and cause loss of access to older content on web pages.

Increased time elapsed since funding, while decreasing link accessibility, does not have a simple negative influence on accessibility and sustainability. Links and dependencies can become outdated, as shown above; however, additional time may allow for CDI project deliverables to be completed or incorporated into another project. Also, finding additional funding or connecting with other groups to carry on the project may occur over several years following cessation of CDI project funding. We did not normalize for time elapsed since funding from 2010 to the present, because multiple time-related factors may have been present. Instead, we excluded projects completed after 2016 because they were too close in time to the analysis to demonstrate meaningful sustainability.

There were many reasons for outputs not being sustained on an individual-level. These included: tools or plug-ins not kept up to date with current versions of software on which there is a dependency; projects subsumed into a larger project and subsequently evolving, so that there is no longer any evidence of the original output (but this could be considered as community-level sustainability because a capacity to do something was preserved); virtual training modules becoming out of date when new policy is implemented, and thus being removed from the internet; projects in which the type of output does not lend itself to sustainability, such as a one-time workshop; a web service that is no longer maintained because it was never an operational system; projects that never delivered a working application, for one of the following reasons: policies changed, which made the development plan no longer feasible; an algorithm did not work as expected, resulting from limitations in dealing with transfer, analysis, or storage of the required data volumes; projects did not provide specific links to their outputs to the CDI facilitators; turnover of staff after the original project period; new funding priorities; setbacks in schedule because of issues found with existing data or previous data analysis; and external collaborators not prioritizing the project as intended.

Deeper analysis into some of the sustainability influences may contribute more insights into the nuances between accessibility and sustainability. For example, for use of code repositories, we could investigate metrics such as clones, forks, commits, and issues, to better understand use and maintenance of delivered source code. Such an analysis may yield different conclusions about project sustainability type achieved.

### Project design

Two of the sustainability influences fall in the category of project design—outputs modified, and code repository used. Resources are available to help investigators with project design of web accessible projects, such as the Science Gateways Community Institute (https://sciencegateways.org/about/service-areas, [5]). We found that many projects did not have time during the seed-funded project period to complete tasks such as communicating value of the completed project to relevant communities. One principal investigator noted: *Making the registry known and making it easily usable and reliable are key to sustainability; yet*, *the need to explore feasible options for continued use and developing awareness takes resources as well*. Training, outreach, finalizing code and making it publicly available, documentation, and usability testing are activities that are seldom completed in the short project time frame. If a project champion is available to search for additional opportunities to sustain project outcomes, the communication of value and opportunities for sustainment could happen several years after the initial project funding. One example is the Adopt-a-Pixel mobile application funded in 2014 [33]—it was completed and maintained on a USGS website, but the principal investigators reflected that use was low and they did not have resources for outreach. In 2018, NASA, an original project collaborator, brought the concept of the application under its Globe Observer mobile app [34, 35] as the Land Cover module. This transition gave the application exposure to an existing citizen science application with over 118,000 user accounts [36].

#### The role of persistent identifiers

We found that certain types of outputs are more likely to be sustained, such as those that require PIDs. Findable and accessible outputs with PIDs supply the raw materials for a new project to build upon. The output types associated with PIDs and thus those that are more likely to be accessible in the long-term are publications, data releases, and source code. In our study, 12 of 16 publications delivered by CDI projects have PIDs, which are commonly associated with longevity and accessibility. Assigning PIDs to publications (USGS uses DOIs) is a standard practice among most journal publishers [37, 38]. This was not the case in the past, and of the four publications without DOIs at the time of this analysis, two are from 2010 when USGS did not yet assign DOIs to its series publications. (USGS has since back-assigned DOIs to all USGS publications.) Another publication without a PID is a formal, but un-peer-reviewed publication, and the last is a paper in an open source journal that does not assign DOIs.

Data releases are datasets the USGS has made formally available to the public, per the requirements of the USGS Public Access Plan [20] and USGS Fundamental Science Practices policies (https://www.usgs.gov/about/organization/science-support/survey-manual/5028-fundamental-science-practices-review-and) [23]. Eight of eleven data releases produced by CDI projects have DOIs. USGS data releases produced since October 2016 are required to have a DOI. Three data releases produced previous to October 2016 do not have DOIs, but instead have web URLs that may not be persistent.

One code repository has a PID. The use of PIDs to uniquely identify code repositories will increase through time because of the recent implementation of the USGS software release policy. For formal code releases, such as those supporting scientific manuscripts, the USGS software release policy now requires a persistent identifier, that the code exist on federally-owned servers, and that the code has a simple metadata record following the Federal Source Code Policy (https://www.usgs.gov/about/organization/science-support/survey-manual/im-osqi-2016-01-review-and-approval-software).

As PIDs become more widespread, we can expect the accessibility, and hopefully the reuse and sustainability, of CDI project outputs to increase. As noted in the results, the sustainability influence with the biggest increase from the group of all projects to the group of projects achieving individual-level and organization-level sustainability was “outputs modified.” This implies that building upon previous outputs in more than a single project cycle leads to greater use and possibility of operationalization in an organization.

### Organizational setting and community environment

The sustainability influence of workforce stability, while widely present, was notably absent in a few cases. Usually, departure of important project personnel leads to decreased achievement of original goals or sustainment of products. As one principal investigator remarked: *If initial project leadership leaves*, *their replacements might not be committed to project success*. *That’s a difficult scenario to plan for*.

Although not shown with our data, it is commonly thought that collaboration across geography or disciplines appears to make a stronger case for long term sustainability because interest is diversified over a greater number of organizations with different requirements and constraints. Even though evidence of this is lacking in our sustainability influence data (Fig 8), studies from the health science field [39] and comments from CDI investigators support this. As mentioned by one investigator in their response: *In hindsight it may have worked better to build it in cooperation with another funding (even if minor) entity and allow them to provide a small funding amount for upkeep beyond the initial year*.

Goring et al. [40] describe how interdisciplinary research has up-front costs (disciplinary conflict, academic freedom, loss of credit, lower publication impact) that must be met before the benefits and results of the collaborations are realized. Therefore, the short-term projects studied here may not show any benefits in their own timeframe. The benefits may be seen only in longer-term collaboration patterns and outputs. The increased impact of research conducted by diverse teams (measured by bibliometrics) and the longer timeline required to realize project benefits are discussed in the science of team science literature (e.g., [39, 41, 42, 43]).

Each organization, such as the USGS, has its own policies, requirements, and constraints on data publication and accessibility. These requirements may have a positive effect on a product’s sustainability if the product helps to meet a policy need. For example, if all data releases must have an accompanying metadata record in a particular format, then a tool that helps to create those metadata records will have high demand, more users, and programs that may be willing to operationalize the tool. One might assume that all projects that have the integration with policy sustainability influence would become organizationally sustained, but we do not see this in our data. Additionally, policies related to data or software have changed during the timeframe of this study, and software technologies may change even faster, which may explain why we do not see the correlation between policy integration and organization-level sustainability.

At the USGS, data supporting publications must have machine-readable metadata, and additionally, data and metadata must be reviewed by peers before release [44]. While this policy becomes a sustainability influence by creating demand for tools to meet requirements, the data policies can alternatively restrict the posting of data or software when CDI project teams lack sufficient resources and motivation to complete the data release process required by policy during the short project duration. Products that are not formally released become less findable and accessible, sometimes existing on restricted-access web pages instead of being indexed in a publicly searchable repository.

Government policy affects the dissemination of products in other ways. The physical and biological sciences, including the geosciences, have moved toward the use of preprint servers recently to facilitate DOI creation and version control for manuscripts that have not gone through official peer review [45]. However, USGS authors are disallowed from placing draft manuscripts where they are available to the public, owing to the 2004 Office of Management and Budget (OMB) Peer Review Bulletin [44], which directs federal agencies to ensure their research has been peer reviewed prior to public dissemination. Resultant USGS policy requires interpretive scientific information products receive peer review and Bureau approval before dissemination to the public [46]. So far, the rise of preprint server popularity and its benefits in disseminating useful work quickly in the non-federal scientific community has not changed this policy. Conversely, government policy can increase access to peer-reviewed and published work by requiring public access to work produced using public funds. The USGS Public Access plan specifies that journal articles authored by USGS employees are “made available free-of-charge from the USGS Publications Warehouse no later than 12 months after publication.” [20]. This means that work initially published behind a paywall becomes publicly accessible at the USGS Publications Warehouse (https://pubs.er.usgs.gov/) in a reasonable amount of time.

### Other considerations: Knowledge transfer, correlation of influences, and sunsetting

Considering only delivered outputs does not take into account the value of knowledge transfer, which is the difficult-to-quantify capacity building of a community. One principal investigator reported the following outcome: *A group of USGS employees learned a better way of working and became leaders in spreading those better methods throughout USGS*. Even if proposed outputs were not achieved, some amount of knowledge was transferred to the CDI community and beyond.

Each funding body must decide how much responsibility it will take on in making the lessons learned accessible and promoting them to potential interested audiences. The CDI stresses that all lessons learned from a project, if shared with the community, are valuable. Shared lessons prevent duplication of effort and increase communication of knowledge across disciplinary or geographic boundaries. The CDI does this by maintaining a publicly available online location (a ScienceBase page) for each project with relevant links, requiring funded project written reports, publishing outcomes in publicly-available official USGS reports, and requiring a presentation to the CDI of lessons learned and challenges faced. The presentation slides and recordings are available to any CDI member, and anyone interested in contributing to the community and supplying a professional email address can join the CDI. Project information is thus fed back to the CDI and USGS for further work or discussion. Therefore, by incorporating the CDI proposals process into a community that promotes sharing and dialogue, the CDI is inherently facilitating knowledge transfer, and is a vehicle for sustaining its funded outputs.

One way our evaluation could be expanded is through network analysis, a process of investigating social structures through the use of networks and graph theory [47]. Network analysis has been applied to other scientific communities and their publications and collaboration structures [47, 48, 49]. To investigate the sustainability of projects, each CDI project would represent a node, and the number of connections to other projects, or the direction of knowledge transfer between projects, could be compared with the persistence of projects over time. Another way to expand the analysis would be to perform multivariate analysis to test for correlations among the influences [8], or to give different weights to the influences depending on their importance.

Finally, we consider the question: should outputs from all projects be sustained? With time, some capabilities become irrelevant, or other widely-available tools may accomplish a task that used to be custom built. This leads us to offer the viewpoint that technical outputs should be reviewed for relevance and sunsetted when appropriate. Over the time window of this study, web technologies have grown and faded in utility. Particularly for the short-term projects examined here, community knowledge advancement through documented lessons is highly valued whether or not the project is fully sustained. Public code repositories and documentation of the project’s findings, preferably linked to persistent identifiers, and continued communication about the project’s outputs, help to achieve the goal of community advancement.

## Summary and conclusion

This study yields lessons for those applying for short term grants and guidance for those who lead programs that provide seed-funding, using the example of a community that funds Earth science informatics projects. We consider the lessons shared by project personnel with the CDI community as most instructive for practitioners that are entering the Earth science informatics field from a pure science background, because considerations for the sustainability of informatics products such as web applications and software differ from those for traditional scientific publications. The 60 projects funded by the USGS Community for Data Integration from 2010–2016 have produced many accessible outputs, with an increasing amount using persistent identifiers. The projects were evaluated for seven relevant sustainability influences drawn from other studies in other fields: outputs modified, code repository used, champion present, workforce stability, support from other organizations, collaboration/partnerships, and integration with policy, and also were categorized into groups that met individual-level sustainability and organization-level sustainability. Projects that achieved high sustainability had higher numbers of sustainability influences, but our mapping did not show a pattern of specific influences that differed from the full pool of projects. However, valuable considerations can be derived from our study of sustainability based on influences for both individuals proposing projects for funding, and for funders who provide seed funding to project proposals.

Lessons for individuals proposing seed-funded projects:

- In project design, plan for the sustainability influences shown here: outputs modified/modifiable, code repository used, champion present, workforce stability, support from other organizations, collaboration/partnerships, and integration with policy
- Look for collaborators to diversify the type of organizations supporting the project
- Create contingency plans for critical personnel departures
- When possible, use persistent identifiers for products
- Commit resources and time to communicate the value of outputs after they are completed
- Build upon previous results and make results reusable
- If trying to learn about a previous project, contact the project personnel for information or resources that may not be readily available or public. Not all outputs become public, especially if there are many organizational requirements that restrict public release. The project personnel or the funding body facilitators may have access to more outputs and information than what is available online.

Guidance for those who implement funding programs and seek to enhance sustainability:

- Decide on the amount of responsibility to be taken on for communicating value of project outputs and commit the requisite amount of resources.
- Explicitly point to sustainability influences in proposal guidance and make them significant criteria for evaluation. For example, encourage use of persistent identifiers even if they are not required by data policy.
- Create easy access to products from past funding so that others can build upon them, which provides efficiency for short-term funded efforts.
- Projects that do not deliver what was initially proposed may develop partial outputs or new collaborations that lead to future useful and popular outputs. Therefore, consider that a measurement of only sustained concrete outputs is not the only measure of utility or success, and try to highlight and communicate the lessons learned.
- Provide guidance on organizational policies that affect how products are released and sustained to help project teams succeed; for example, communicate requirements such as peer review requirements before official release.

This evaluation of an annual seed-funding proposals process provided an informative way to investigate funding outcomes of Earth science informatics projects. This process identified required modifications to CDI guidance and selection of proposals that will increase the likelihood of sustained outputs.

## Supporting information

S1 Table Projects funded by the USGS community for data integration between 2010 and 2016. Further details on each project can be found at http://www.usgs.gov/cdi.
(PDF)

S2 Table All sustainability influences from references 7–10. This table lists the sustainability influences examined from references 7–10. For sustainability influences not used in our study, an explanation is given. Other funding organizations may choose to include some of the influences that we did not.
(PDF)

S1 File Examples of community for data integration project outputs. Additional examples of CDI output types to add to those described in the main body of the paper.
(PDF)

S1 Text Email to principal investigators. Text of the email sent to all past Community for Data Integration project principal investigators to ask for information about their project outputs.
(PDF)

## References

[1] Maron N. A guide to the best revenue models and funding sources for your digital resources. Ithaka S+ R. 2014, Available from: http://www.sr.ithaka.org/wp-content/uploads/2015/08/Jisc_Report_032614.pdf.

[2] Signell RP, Barker C, Dalyander P, Hunt C, Knudsen R, Ring K, et al Evaluating a new open-source, standards-based framework for web portal development in the geosciences: ScienceBase 2016 Available from: https://www.sciencebase.gov/catalog/item/56d87a7de4b015c306f6cfcf.

[3] Geoweaver: a web-based prototype system for managing compound geospatial workflows of large-scale distributed deep networks [Internet].[cited 2019 Aug 15]. Available from: https://github.com/ESIPFed/Geoweaver.

[4] Sediment Experimentalists Network [Internet].[cited 2019 Aug 15]. Available from: https://www.earthcube.org/group/sen.

[5] Gesing S, Wilkins-Diehr N, Dahan M, Lawrence K, Zentner M, Pierce M, et al Science gateways: the long road to the birth of an institute, Proceedings of the 50th Hawaii International Conference on System Sciences, 2017 Available from: http://hdl.handle.net/10125/41919.

[6] Hsu L, Langseth ML. Community for Data Integration 2017 annual report. U.S. Geological Survey; 2018 10.3133/ofr20181110

[7] Shediac-Rizkallah MC, Bone LR. Planning for the sustainability of community-based health programs: conceptual frameworks and future directions for research, practice, and policy, Health Education Research 1998;13(1):87–108. 10.1093/her/13.1.87 10178339

[8] Scheirer MA. Is Sustainability Possible? A Review and Commentary on Empirical Studies of Program Sustainability, American Journal of Evaluation, 2005 10.1177/1098214005278752

[9] Stirman SW, Kimberly J, Cook N, Calloway A, Castro F, Charns M. The sustainability of new programs and innovations: a review of the empirical literature and recommendations for future research, Implementation Science, 2012 10.1186/1748-5908-7-17 22417162 PMC3317864

[10] Stewart CA, Almes GT, and Wheeler BC (eds). Cyberinfrastructure Software Sustainability and Reusability: Report from an NSF-funded workshop. Published by Indiana University, Bloomington, IN 2010 Available from http://hdl.handle.net/2002/6701.

[11] Wernert J, Wernert EA, and Stewart CA. Models for Sustainability for Robust Cyberinfrastructure Software—Software Sustainability Survey, Indiana University, Bloomington, IN PTI Technical Report PTI-TR13-007. 2013 Available from: http://hdl.handle.net/2022/17313.

[12] Arp LG, Forbes M, Cartolano RT, Cramer T, Kimpton M, Skinner K, et al It Takes a Village: Open Source Software Sustainability. Columbia University Academic Commons, 2018 10.7916/D89G70BS

[13] Paskin N. Digital Object Identifiers for scientific data. Data Science Journal. 2006; 12–20. 10.2481/dsj.4.12

[14] Wilkinson MD, Dumontier M, Aalbersberg IJ, Appleton G, Axton M, Baak A, et al The FAIR Guiding Principles for scientific data management and stewardship. Scientific data, 3 2016 10.1038/sdata.2016.18 26978244 PMC4792175

[15] Hsu L, Hutchison VB, and Langseth ML, Data on the Deliverables, Sustainability, and Collaboration of Community for Data Integration Projects from 2010–2016: U.S. Geological Survey, 2019. 10.5066/P9V3XDY6

[16] Holl S. U.S. Geological Survey Community for Data Integration—NWIS Web Services Snapshot Tool for ArcGIS: U.S. Geological Survey Fact Sheet 2011–3141, 2011. 10.3133/fs20113141

[17] Ignizio DA, O’Donnell MS, Talbert CB. Metadata wizard—An easy-to-use tool for creating FGDC–CSDGM metadata for geospatial datasets in ESRI ArcDesktop: U.S. Geological Survey Open-File Report, 2014–1132. 2014 10.3133/ofr20141132

[18] Rosemartin A, Langseth ML, Crimmins TM, Weltzin JF. Development and release of phenological data products—A case study in compliance with federal open data policy: U.S. Geological Survey Open-File Report 2018–1007. 2018 10.3133/ofr20181007

[19] Guy M, Earle P, Horvath S, Turner J, Bausch D, Smoczyk G. Social Media Based Earthquake Detection and Characterization, 2014 KDD Workshop on Learning about Emergencies from Social Information (KDD-LESI 2014), New York City, USA, August 24, 2014. Available from: https://a5c39290-a-62cb3a1a-s-sites.googlegroups.com/site/kddlesi2014/kddlesi2014_proceedings.pdf.

[20] U.S. Geological Survey. Public Access to Results of Federally Funded Research at the U.S. Geological Survey: Scholarly Publications and Digital Data, 2016. Available from: https://www2.usgs.gov/quality_integrity/open_access/downloads/USGS-PublicAccessPlan-APPROVED-v1.03.pdf.

[21] Henkel HS, Hutchison VB, Chang MY, Zolly L, Uribe R, Faust T. Data Management Website, ScienceBase, 2012 Available from: https://www.sciencebase.gov/catalog/item/523b7415e4b08cabd166d20f.

[22] Faundeen JL, Burley TE, Carlino JA, Govoni DL, Henkel HS, Holl SL, et al United States Geological Survey Science Data Lifecycle Model: U.S. Geological Survey Open-File Report 2013–1265. 2013, 10.3133/ofr20131265

[23] Fundamental Science Practices Advisory Committee. U.S. Geological Survey Fundamental Science Practices: U.S. Geological Survey Circular 1367, 2011.

[24] Faundeen JL, Everette AL, Zolly L, Cross VA, Kemp SK, Galvan S, et al Developing a USGS Legacy Data Inventory to Preserve and Release Historical USGS Data. ScienceBase, 2016 Available from: https://www.sciencebase.gov/catalog/item/56d87142e4b015c306f6cf9b.

[25] Chase KJ, Bock AR, Sando R. Sharing our data—An overview of current (2016) USGS policies and practices for publishing data on ScienceBase and an example interactive mapping application: U.S. Geological Survey Open-File Report 2016–1202, 2017, 10.3133/ofr20161202

[26] Gordon J, Chkhenkeli N, Govoni D, Lightsom F, Ostroff A, Schweitzer P, et al A case study of data integration for aquatic resources using semantic web technologies: U.S. Geological Survey Open-File Report 2015–1004, 2015 10.3133/ofr20151004

[27] Plale B, Kouper I. The centrality of data: data lifecycle and data pipelines. In Data Analytics for Intelligent Transportation Systems 2017 1 1 (pp. 91–111). Elsevier. 10.1016/B978-0-12-809715-1.00004-3

[28] Gundersen LC. Scientific integrity and ethical considerations for the research data life cycle. Scientific Integrity and Ethics: With Applications to the Geosciences. 2017 10 17:133–53. 10.1002/9781119067825.ch9

[29] Thieler ER, Zeigler SL, Winslow LA, Hines MK, Read JS, Walker JI. Smartphone-based distributed data collection enables rapid assessment of shorebird habitat suitability. PloS one. 2016 11 9;11(11):e0164979 10.1371/journal.pone.0164979 27828974 PMC5102412

[30] Han T, Rukhlov AS, Riddell JM, Ferbey T. A skeleton data model for geochemical databases at the British Columbia Geological Survey. 2018 Geological Fieldwork 2018, British Columbia Ministry of Energy, Mines and Petroleum Resources, British Columbia Geological Survey Paper 2019–01:125–136.

[31] Esri Product Lifecycle Support Policy, 2019 [cited Aug 14, 2019]. Available from https://downloads2.esri.com/support/TechArticles/Product-Life-Cycle.pdf.

[32] Silberschatz A, Gagne G, Galvin PB. Operating system concepts. Wiley; 2018 1 18.

[33] Earth Resources Observation and Science Center, Adopt a Pixel, 2019, 10.5066/F7VM4B7C

[34] Global Learning to Benefit the Environment (GLOBE) Data User Guide, 2019, version 1.0, www.globe.gov.

[35] Global Learning and Observations to Benefit the Environment (GLOBE) Program, 2019 Jul 26, https://www.globe.gov/globe-data.

[36] Hayden L, Taylor J, Robles MC. GLOBE: Connecting to Community of Observers Directly to NASA Satellites [Education]. IEEE Geoscience and Remote Sensing Magazine 2019;7(1):98–99. 10.1109/MGRS.2019.2891930

[37] DeRisi S, Kennison R, Twyman N. The What and Whys of DOIs, PLoS Biol 2003;1(2):e57 10.1371/journal.pbio.0000057 14624257 PMC261894

[38] Boudry C, Chartron G. Availability of digital object identifiers in publications archived by PubMed, Scientometrics 2017;110:1453–1469. 10.1007/s11192-016-2225-6

[39] Hall KL, Vogel AL, Huang GC, Serrano KJ, Rice EL, Tsakraklides SP, Fiore SM. The science of team science: A review of the empirical evidence and research gaps on collaboration in science. American Psychologist. 2018 5;73(4):532 10.1037/amp0000319 29792466

[40] Goring SJ, Weathers KC, Dodds WK, Soranno PA, Sweet LC, Cheruvelil KS, et al Improving the culture of interdisciplinary collaboration in ecology by expanding measures of success. Frontiers in Ecology and the Environment 2014;12:39–47, 10.1890/120370

[41] Stokols D, Hall KL, Taylor BK, Moser RP. The science of team science: overview of the field and introduction to the supplement. American journal of preventive medicine. 2008 8 1;35(2):S77–89. 10.1016/j.amepre.2008.05.007 18619407

[42] Stokols D, Misra S, Moser RP, Hall KL, Taylor BK. The ecology of team science: understanding contextual influences on transdisciplinary collaboration. American journal of preventive medicine. 2008 8 1;35(2):S96–115. 10.1016/j.amepre.2008.05.003 18619410

[43] Hall KL, Vogel AL, Stipelman BA, Stokols D, Morgan G, Gehlert S. A four-phase model of transdisciplinary team-based research: goals, team processes, and strategies. Translational behavioral medicine. 2012 10 25;2(4):415–30. 10.1007/s13142-012-0167-y 23483588 PMC3589144

[44] Office of Management and Budget (OMB), M-05-03 Final Information Quality Bulletin for Peer Review. [cited 2019 Aug 12] Available from: https://georgewbush-whitehouse.archives.gov/omb/memoranda/fy2005/m05-03.pdf.

[45] Narock T, Goldstein EB, Jackson CA, Bubeck AA, Enright AML, Farquharson JI, et al Earth science is ready for preprints, Eos, 2019;100, 10.1029/2019EO121347

[46] U.S. Geological Survey Manual 502.4—Fundamental Science Practices: Review, Approval, and Release of Information Products. [cited 2019 Aug 12] Available from: https://www.usgs.gov/about/organization/science-support/survey-manual/5024-fundamental-science-practices-review-approval.

[47] Otte E, Rousseau R. Social network analysis: a powerful strategy, also for the information sciences. Journal of Information Science 2002;28(6):441–453. 10.1177/016555150202800601

[48] Zoss AM, Börner K. Mapping interactions within the evolving science of science and innovation policy community. Scientometrics 2011;91(2):631–44. 10.1007/s11192-011-0574-8

[49] Hicks DJ, Coil DA, Stahmer CG, Eisen JA. Network analysis to evaluate the impact of research funding on research community consolidation. PLoS ONE 2019;14(6):e0218273 10.1371/journal.pone.0218273 31211808 PMC6581276

