# Supplementary material for: Measuring sustainability of seed-funded earth science informatics projects
Source: PLoS One. 2019 Oct 23;14(10):e0222807. doi: 10.1371/journal.pone.0222807 (PMC6808333; doi:10.1371/journal.pone.0222807)
Supplement: S1 Table — Further details on each project can be found at http://www.usgs.gov/cdi. (PDF) [file pone.0222807.s001.pdf]

S1 Table. Projects funded by the USGS Community for Data Integration between 2010 and 2016.

Further details on each project can be found at http://www.usgs.gov/cdi.

ID	Title
FP10-BD	Geo Data Portal
FP10-BS	Added to the National Map: Web Services for the National Hydrography Dataset, National Elevation Dataset and National Land Cover Dataset
FP10-HS	NWIS Web Services Snapshot for ArcGIS
FP10-KT	Data Upload, Registry and Access Project (a capability of ScienceBase)
FP11-FJ	Science Data Lifecycle Model
FP11-HV1	Data Management Website
FP11-HV2	Data Management Training and Education
FP11-HV3	Data Management Workshop
FP11-KT1	Enhanced Metadata Capability for CBI Metadata Tool-ScienceBase
FP11-KT2	FGDC-ISO Remapping for CBI Metadata Tool-ScienceBase
FP12-AJ	Implementing an OpenLayers HTML5 Mapping Library
FP12-BD	Geo Data Portal - ScienceBase/GDP, Python port for GDP
FP12-BT	USGS Science Center Adaptable Data Management Plan Implementation and Framework
FP12-DR	Expand the National Map Save As/Open In to USGS Wide
FP12-GJ	Semantic Technologies for Integrating USGS Data
FP12-HM	USGS Citizen Science Workshop
FP12-MD	Mobile application to collect national consistent data of fish passage barriers in the United States
FP12-MD2	Citizen Science Observation Platform - Using Curated Twitter and GeoRSS Enabled Feeds
FP12-SJ	Facilitating Knowledge Integration with a Monitoring Protocol Registry
FP12-SL	USGS Mobile Applications Development Support Framework
FP13-BA	Evaluation of downscaled General Circulation Model (GCM) output for current conditions and associated error in simulated runoff for CONUS
FP13-BD	Establishing the Land Use Land Cover Geo Data Portal and Supporting Data Services
FP13-EA	Mining the USGS Data Landscape
FP13-FG	Development of Enhanced Feature Recognition Software for the Extraction of Mine Features from USGS Topographic Maps
FP13-FL	Networking the California Climate Commons with the USGS Geo Data Portal

FP13-HC	National Land Cover Database Visualization and Information Tool
FP13-HS	myScience: USGS Citizen Science Project Discovery & Public Engagement Web Application
FP13-HV	Data Management Workshops for USGS: Let the Culture Change Begin
FP13-ID	Metadata Wizard: An Easy-to-Use Tool for FGDC Metadata Creation in ESRI's ArcGIS Software
FP13-SD	USGS Protocol Library: An Implementation Based on the National Environmental Methods Index
FP14-DR	Summarization of National NEXRAD Data for use in Biological Applications
FP14-EL	North American Bat Data Integration
FP14-FP	NASWeb API Web Services Access to the Nonindigenous Aquatic Species Database
FP14-GM	Characterization of Earthquake Damage and Effects Using Social Media Data
FP14-LF	Use of Controlled Vocabularies in USGS Information Applications: Requirements Analysis for Automated Processes and Services (Bureau-wide Application)
FP14-LJ	Online Merging and Gridding of Topographic and Bathymetric Data Sources
FP14-LR	Adopt a Pixel - Data Infrastructure
FP14-SR	Geographic Searches for USGS Publications (Bureau-wide Application)
FP14-SS	Portable ISO 19115-2 Open Source Developer's Toolkit (Bureau-wide Application)
FP15-BD	The 'Digital Grain Size' Web and Mobile-Computing Application
FP15-DJ	National Dam Removal Database: A living database for information on dying dams
FP15-GT	Geocaching Natural Features - Applying Game Mechanics to Citizen Science Data Collection
FP15-LJ	Making Unmanned Aircraft System (UAS) Data Available to USGS Scientists and the Public
FP15-LR	Web-enabled Visualization and Access of Value-added Disaster Products
FP15-RP	Standards-based Integration and Delivery of USGS and EPA STORET Biomonitoring Data via the Water Quality Data Portal
FP15-SC	Integration of Land Cover Trends Field Photography with an Online Map Service
FP15-WL	sbtools: An R package for ScienceBase
FP16-CK	Development of Recommended Practices and Workflow for Publishing Digital Data through ScienceBase for Dynamic Visualization
FP16-EA	Developing a USGS Legacy Data Inventory to Preserve and Release Historical USGS Data
FP16-GM	Crowd-Sourced Earthquake Detections Integrated into Seismic Processing
FP16-JJ	A data management and visualization framework for community vulnerability to hazards
FP16-MS	Hunting Invasive Species with HTCondor: High Throughput Computing for Big Data and Next Generation Sequencing
FP16-NJ	Data Management Training Clearinghouse
FP16-PT	Birds and the Bakken: Integration of oil well, land cover, and species distribution data to inform conservation in areas of energy development

FP16-SE	Integration of National Soil and Wetland Datasets: A Toolkit for Reproducible Calculation and Quality Assessment of Imputed Wetland Soil Properties
FP16-SJ	A web-based application for the management and visualization of land-use scenario data
FP16-SR	Evaluating a new open-source, standards-based framework for web portal development in the geosciences
FP16-TC	Facilitating the USGS Scientific Data Management Foundation by integrating the process into current scientific workflow systems
FP16-WD	National Stream Summarization: Standardizing Stream-Landscape Summaries
FP16-WJ	Integration of Phenological Forecast Maps for Assessment of Biodiversity: An Enterprise Workflow

Supporting information for Hsu, Hutchison, and Langseth, Measuring sustainability of seed-funded Earth science informatics projects.
