# Supplementary material for: Measuring sustainability of seed-funded earth science informatics projects
Source: PLoS One. 2019 Oct 23;14(10):e0222807. doi: 10.1371/journal.pone.0222807 (PMC6808333; doi:10.1371/journal.pone.0222807)
Supplement: S2 Table — This table lists the sustainability influences examined from references 7–10. For sustainability influences not used in our study, an explanation is given. Other funding organizations may choose to include some of the influences that we did not. (PDF) [file pone.0222807.s002.pdf]

S2 Table. All sustainability influences from references 7-10.

This table lists the sustainability influences examined from references 7-10. For sustainability influences not used in our study, an explanation is given. Other funding organizations may choose to include some of the influences that we did not.

Reasons for not using a sustainability influence in our study:

- **Difficult to quantify for all CDI projects**. Presence of the influence cannot be determined from the proposal text or final report. Example: Project effectiveness. Is the project (perceived as) effective?
- **The value is similar for almost all CDI projects.** Example: project duration. Almost all CDI projects (with the exception of three in 2014-2015) are funded for one fiscal year.
- **CDI Output type can be used as a similar factor.** Project type. Example given in the reference from the health field is "preventative vs. curative." Those categories do not apply for CDI projects, but CDI output types (e.g., software vs. data release) provide a useful categorization.
- **Not applicable to most/many CDI projects.** Example: Low cost – uses volunteers. The example from implementation of health programs does not apply to our informatics projects. (Even citizen science projects would require non-volunteers to develop and implement.)
- **Hard to evaluate without a definition**. One of the references did not provide definitions of its sustainability influences, and therefore not all influences are well-described enough to evaluate here.
- **Part of CDI selection criteria, so this influence wouldn't be helpful for distinguishing among funded projects.** Some influences are critical elements of the scoring criteria used to select CDI projects for funding. Therefore, almost all CDI projects possess the influence and there is little variability in our dataset.

Influence	Original definition	Used here?	If not used, why?	Note
**[7] Shediac-Rizkallah, MC, Bone LR. Planning for the sustainability of community-based health programs: conceptual frameworks and future directions for research, practice, and policy, Health Education Research 1998;13(1):87-108.<br>Table II. Guidelines for sustainability planning**				
Project negotiation process	Are project approaches and goals discussed with recipient community members, as equal partners? Are the needs of the community driving the	No	Difficult to quantify for all CDI projects	

	program or those of external donor agencies and technical experts? Is a negotiation or consensus-building process in place to reach a compromise for addressing everyone's (including donors, community, technical experts) needs?			
Project effectiveness	Is the project (perceived as) effective? Is it visible? What are the (desirable and undesirable) secondary effects of the program?	No	Difficult to quantify for all CDI projects	
Project duration	What is the project's grant period (number of years in operation)? Is it a new project or is it an existing program that is acquiring additional funds?	No	The value is similar for almost all CDI projects	
Project financing	What are the sources of funds for the program (internal, external, a mixture)? What are the community's local resources? Can the community afford the program (e.g. is it able to pay maintenance and recurrent costs)? How much are community members willing/able to pay for services? What strategies are in place to facilitate gradual financial self- sufficiency?	No	Difficult to quantify for all CDI projects	
Project type	What type of project is it (e.g. preventive versus curative)?	No	CDI Output type can be used as a similar factor	
Training	Does the project have a training component (professional or para-professional)?	No	Difficult to quantify for all CDI projects	
Institutional strength	What organization will be implementing the program? How mature (developed, stable, resourceful) is this organization? Is it likely to provide a strong organizational base for the program?	No	Difficult to quantify for all CDI projects	
Integration with existing programs/services	Is the program vertical (categorical) or is it a horizontal (comprehensive or integrated) program? Are goals, objectives and approaches pre-specified or are they adapted to the local population and setting and over time? Is the program integrated into the standard operating practices of its host organization? Is the mission of the program compatible with the mission and activities of its host organization? Is the	Yes		"Integration with Policy"

	implementing organization the recipient of program funds or is there an intermediary organization?			
Program champion/leadership	Is there a program champion? What are his/her attributes? If not, can one be identified/ nurtured so that he/she may serve as an advocate for the continuation of the program? Is the program endorsed from the top? How well is it supported?	Yes		"Champion present"
Socioeconomic and political considerations	Socioeconomic and political considerations. How favorable is the general socioeconomic and political environment for the sustainability of the program to be a realistic goal?	No	Difficult to quantify for all CDI projects	
Community participation	What is the level of community participation? What is the depth (amount) of involvement? What is the range of involvement (types of activities)?	No	Difficult to quantify for all CDI projects	
**[8] Scheirer MA. Is Sustainability Possible? A Review and Commentary on Empirical Studies of Program Sustainability, American Journal of Evaluation, 2005. doi:10.1177/1098214005278752. In section "Factors Related to Extent of Sustainability"**				
% of Sites Sustained		No	Not applicable to most CDI projects.	
Program Modifiable	programs that were modifiable at the local level were more likely to be sustained	Yes		"Outputs modified"
Low Cost: Uses Volunteers	the use of volunteers or other low-cost ways of delivering services as a key strategy for sustainability	No	Not applicable to most CDI projects.	
Positive Evaluation	the use of evaluation data as an important vehicle for gaining support needed for continuation	No	Difficult to quantify for all CDI projects	
Champion Present	a program champion, sometimes the executive director. This is a person who is strategically located to have access to upper management as well as influence on, or control over, day-to-day program operations. The champion often enthusiastically advocated for the needs of the program, particularly to help secure resources for its continuation.	Yes		"Champion present"
Strong Existing Capacity	the strength of existing organizational capacity as a key aspect influencing sustainability	No	Part of CDI selection criteria, so this	

			influence wouldn't be helpful for distinguishing among funded projects	
Fit with Mission/Tasks	the “fit” of the new program within the existing organizational mission and/or its standard operating procedures as a key influence on sustainability. Project activities that could be “sold” as contributing to the organization’s goals were more likely to receive internal sup- port and even resources that allowed them to be sustained. Furthermore, project activities that could readily fit into existing tasks and procedures were more likely to have the support of operating staff members. However, it is also possible that some of these were continuations of activities that the organization had started up before the “new” funding for the project studied and would have contin- ued even in the absence of that specific source of funding.	No	Part of CDI selection criteria, so this influence wouldn't be helpful for distinguishing among funded projects	"Integration with Policy"
Perceived Benefits to Staff Members/Clients	when staff members or key stakeholders could perceive benefits to themselves and/or to clients, the program was more likely to be sustained	No	Part of CDI selection criteria, so this influence wouldn't be helpful for distinguishing among funded projects	
Longer Time Period	Most studies waited for a meaningful amount of time to elapse before examining sustainability: At least 13 studies contacted the original sites at least 2 years after external funding had ended (if there was external funding) or had a varied length of time after funding ended before data collection. There is no commonly accepted time point for defining when a program is “sustained.”	No	We did not assess sustainability for projects less than two years out to give projects enough time to reach sustainability	
Support From Other Organizations	support from other organizations in the environ- ment, for example, for in-kind resources such as expert advice in fund-raising, for political support, or to help mobilize clients to advocate for new funding	Yes		"Collaboration/Partnership"

Type of Funding	funding from other sources, particularly the availability of a larger number of funding sources or the transfer of support to local governmental sources.	Yes	Merged with "support from other organizations"	"Support from other organizations"
**[9] Stirman SW, Kimberly J, Cook N, Calloway A, Castro F, Charns M. The sustainability of new programs and innovations: a review of the empirical literature and recommendations for future research, Implementation Science, 2012. doi:10.1186/1748-5908-7-17.<br>Table 2. Influences on sustainability**				
Ability to be modified/modifications made	no definitions provided	Yes		"Outputs modified"
Effectiveness or benefit		No	Part of CDI selection criteria, so this influence wouldn't be helpful for distinguishing among funded projects	
Ability to maintain fidelity/ integrity		No	Hard to evaluate without a definition	
Climate		No	Difficult to quantify for all CDI projects	
Culture		No	Hard to evaluate without a definition	
Leadership		No	Difficult to quantify for all CDI projects	
Setting characteristics (structure; policies)		No	The value is similar for almost all CDI projects	
System/policy change		No	The value is similar for almost all CDI projects	
Champions (internal or external)		Yes		"Champion present"
Funding		No	The value is similar for almost all CDI projects	
Workforce (staffing, attributes)		Yes		"Workforce stability"
Resources		No	Hard to evaluate without a definition	
Community/stakeholder support/ involvement		No	Part of CDI selection criteria, so this	

			influence wouldn't be helpful for distinguishing among funded projects	
Engagement/relationship building		No	Difficult to quantify for all CDI projects	
Shared decision making among stakeholders		No	Difficult to quantify for all CDI projects	
Adaptation/alignment		No	Hard to evaluate without a definition	
Integration of rules/policies		Yes		
Evaluation and feedback		No	Difficult to quantify for all CDI projects	
Training and education		No	Difficult to quantify for all CDI projects	
Collaboration/partnership		Yes		"Collaboration/Partnership"
Navigating competing demands		No	Difficult to quantify for all CDI projects	
Ongoing support		Yes		"Support from other organizations"
Planning		No	Difficult to quantify for all CDI projects	
**[10] Stewart CA, Almes GT, and Wheeler BC (eds). Cyberinfrastructure Software Sustainability and Reusability: Report from an NSF-funded workshop. Published by Indiana University, Bloomington, IN. 2010. Available from http://hdl.handle.net/2002/6701.<br>Section 6.2 Characteristics of software development teams and processes to create sustainability**				
1. Plan with sustainability in mind				
1a. - project manager with a vision for project and good architecture		No	Difficult to quantify for all CDI projects	
1b. - formal plan for personnel resources, succession plans, ability to port project to another group		Yes		"Workforce stability"
1c. - documented needs analysis and evidence of evaluation of existing codes on which to build		No	Difficult to quantify for all CDI projects; Not	

			applicable to many CDI projects	
1d. - plans for software adoption as well as software development		No	Difficult to quantify for all CDI projects;	Part of CDI selection criteria, however, proposed activities are not always executed, so hard to evaluate.
2. Team acknowledgment of developing software sustainable over the longhaul - which implies that it will someday be sustained by people other than those doing code at any particular time		No	Difficult to quantify for all CDI projects	
3. Use of a specific software development methodology				
3a. - Use of a source control management system		Yes		"Code repository used"
3b. - Open, transparent architecture, easy to comprehend		No	Difficult to quantify for all CDI projects; Not applicable to many CDI projects	
3c. - Evaluation and management of risk, cyberinfrastructure should be lower risk		No	Difficult to quantify for all CDI projects; Not applicable to many CDI projects	CDI seed funding allows for higher risk at lower investment
3d. - Clear documentation of software itself and dependencies on other software		No	Difficult to quantify for all CDI projects	
3e. - Maintain and release software with some sort of schedule or formal release procedure, including management of provenance of code		No	Not applicable to most CDI projects	
3f. - Whenever possible, comply with relevant standards and be vendor agnostic		No	Difficult to quantify for all CDI projects	
4. Develop with clearly documented APIs		No	Not applicable to most CDI projects	

5. Plan to promote adoption as well as development				
5a. - include ongoing evaluation of the software		No	Difficult to quantify for all CDI projects	

Supporting information for Hsu, Hutchison, and Langseth, Measuring sustainability of seed-funded Earth science informatics projects.
