# Supplementary material for: Measuring sustainability of seed-funded earth science informatics projects
Source: PLoS One. 2019 Oct 23;14(10):e0222807. doi: 10.1371/journal.pone.0222807 (PMC6808333; doi:10.1371/journal.pone.0222807)
Supplement: S1 File — Additional examples of CDI output types to add to those described in the main body of the paper. (PDF) [file pone.0222807.s003.pdf]

S1 File. Examples of Community for Data Integration Project outputs.

Additional examples of CDI output types to add to those described in the main body of the paper.

Data Release

Legacy data: Developing a USGS Legacy Data Inventory to Preserve and Release Historical USGS Data
In 1954 researchers at the USGS Great Lakes Science Center conducted 11 research cruises on Lake Michigan during which 779 bathythermographs were cast to collect temperature profile data (temperature at depth). This USGS data release is a digitized set of original bathythermogram print photos and the temperature and depth data the project team collected from them. In addition, because of their historical value and potential future use, this data release includes the cruise logs, which include nautical and research notes.
https://doi.org/10.5066/F7DV1H4B
Sustainability influences: outputs modified, champion present, collaborators/partners, workforce stability

Tsunami Travel Time Maps for Del Norte and Humboldt Counties, CA, reference year 2010
Tsunami travel time maps for Del Norte and Humboldt Counties in California in vector (shapefile) format for both slow and fast walking speeds and for bridges intact and bridges removed.
https://doi.org/10.5066/F7CC0XWN
Sustainability influences: code repository used, workforce stability

Mobile Application

No examples for mobile applications

Presentation

Development of Recommended Practices and Workflow for Publishing Digital Data through ScienceBase for Dynamic Visualization
The project documented processes for USGS scientists to organize and share data using ScienceBase, and provided an example interactive mapping application to display those data. The project team developed an interactive mapping application in R that connects to data on ScienceBase, using Shiny, Leaflet, and sbtools. The project team developed a webinar to share this process with USGS scientists.

https://www.sciencebase.gov/catalog/item/56d87cc4e4b015c306f6cfe1
Sustainability influences: code repository used, champion present, collaborators/partners, integration with policy, workforce stability

Publication

Semantic Technologies for Integrating USGS Data
The project team developed and tested the semantic approach to data integration by focusing on the problem of fish habitat modeling. Effective prediction of the abundance of particular species at particular locations is a primary objective of both ecology and natural resource management. The publication describes production of an information foundation for fish habitat research consisting of a "mashup" of data from multiple USGS data systems that are fragmented among the former USGS Divisions.
https://dx.doi.org/10.3133/ofr20151004
Sustainability influences: outputs modified, champion present, collaborators/partners, workforce stability

Sharing our data—An overview of current (2016) USGS policies and practices for publishing data on ScienceBase and an example interactive mapping application
This report provides an overview of current (2016) U.S. Geological Survey policies and practices related to publishing data on ScienceBase, and an example interactive mapping application to display those data.
https://doi.org/10.3133/ofr20161202
Sustainability influences: code repository used, champion present, collaborators/partners, integration with policy, workforce stability
Citations in the scientific literature:

- Rosemartin AH, Langseth ML, Crimmins TM, Weltzin JF, Development and release of phenological data products—A case study in compliance with federal open data policy. 2018 U.S. Geological Survey Open-File Report 2018–1007. doi: 10.3133/ofr20181007.

Software

Metadata Wizard
This tool is designed as a resource to help geospatial data users with the creation and editing of metadata compliant with the Federal Geographic Data Committee's 'Content Standard for Digital Geospatial Metadata' (FGDC-CSDGM).
www.sciencebase.gov/metadatawizard
Sustainability influences: outputs modified, code repository used, champion present, support from other organizations, integration with policy, workforce stability

Source Code

sbtools
Tools for interfacing R with ScienceBase data services.
https://github.com/USGS-R/sbtools
Sustainability influences: outputs modified, code repository used, champion present, integration with policy, workforce stability
Citations in the scientific literature:

- Anderson E, Chlumsky R, McCaffrey D, Trubilowicz J, Shook KR, Whitfield PH. R-functions for Canadian hydrologists: a Canada-wide collaboration. Canadian Water Resources Journal/Revue canadienne des ressources hydriques. 2019 Jan 2;44(1):108-12.
- Koczkodaj WW, Wolny-Dominiak A. RatingScaleReduction package: stepwise rating scale item reduction without predictability loss. arXiv preprint arXiv:1703.06826. [Preprint]. 2017 [cited 2019 Aug 15]: [20 p.]. Available from: https://arxiv.org/abs/1703.06826.

Web Application

Data Management Training Clearinghouse
The purpose of the Data Management Training (DMT) Clearinghouse project was to increase discoverability, accessibility, and use of digital data management training materials. The project team created an online metadata registry with a publicly available, facet-searchable and browsable inventory of learning resources about data management practices. The team also developed a workflow and mechanism for adding resources to the registry. The registry includes references to community-developed frameworks for data management such as the USGS Science Support Framework and its Science Data Lifecycle Model.
http://dmtclearinghouse.esipfed.org/
Sustainability influences: outputs modified, champion present, support from other organizations, collaborators/partners, integration with policy, workforce stability

Dam Removal Information Portal
The project team created the Dam Removal Information Portal (DRIP) (U.S. Geological Survey, 2015j). This online Web site, powered by CartoDB and using data and services from USGS ScienceBase, currently contains tools for visualization and analysis of georeferenced dam removals and associated scientific studies contained in the American Rivers (2014) and Bellmore and others (2015) databases, respectively.
https://www.sciencebase.gov/drip/

Sustainability influences: outputs modified, code repository used, champion present, support from other organizations, collaborators/partners, workforce stability

Selected citations in the scientific literature:

- Foley MM, Bellmore JR, O'Connor JE, Duda JJ, East AE, Grant GE, Anderson CW, Bountry JA, Collins MJ, Connolly PJ, Craig LS. Dam removal: Listening in. Water Resources Research. 2017 Jul 1;53(7):5229-46. doi: 10.1002/2017WR020457.
- Grigg NS. Global water infrastructure: state of the art review. International journal of water resources development. 2019 Mar 4;35(2):181-205. doi: 10.1080/07900627.2017.1401919.
- McCaffery R, McLaughlin J, Sager-Fradkin K, Jenkins KJ. Terrestrial Fauna are Agents and Endpoints in Ecosystem Restoration Following Dam Removal. Ecological Restoration. 2018 Jun 1;36(2):97-107. doi: 10.3368/er.36.2.97.

Legacy Data Inventory and Reporting System

The project's goal was to expand the USGS contribution to scientific discovery and knowledge by demonstrating a long-term approach to inventorying, prioritizing and releasing to the public the wealth of USGS legacy scientific data. Develop a methodology to evaluate and prioritize USGS legacy data sets based on USGS mission and program objectives and potential of successful release within USGS records management and open data policies.

https://apps.usgs.gov/ldi/content/legacy-data-inventory

Sustainability influences: outputs modified, champion present, collaborators/partners, workforce stability

Web Link

Data Management Website

The USGS Data Management Web Site provides non-prescriptive data management guidance, best practices, tools, and resources in one convenient location. It's intended to complement and support the Survey Manual handbook by providing guidance on how Survey Manual policies can be implemented. It offers a place to share and learn about USGS best practices in data management.

https://www.usgs.gov/products/data-and-tools/data-management

Sustainability influences: outputs modified, champion present, support from other organizations, collaborators/partners, integration with policy, workforce stability

Web Service

NASWeb API Web Services Access to the Nonindigenous Aquatic Species Database
The web services API allows for access to information on occurrence data stored within the NAS database. The national Nonindigenous Aquatic Species (NAS) Database Program serves as a repository for geo-referenced occurrence data on introduced aquatic organisms across the nation. This project designed, tested, and implemented a Web Services API for access to NAS occurrence data.
https://nas.er.usgs.gov/api/documentation.aspx
Sustainability influences: outputs modified, champion present, support from other organizations, collaborators/partners, workforce stability

Supporting information for Hsu, Hutchison, and Langseth, Measuring sustainability of seed-funded Earth science informatics projects.
