# Supplementary material for: Measuring sustainability of seed-funded earth science informatics projects
Source: PLoS One. 2019 Oct 23;14(10):e0222807. doi: 10.1371/journal.pone.0222807 (PMC6808333; doi:10.1371/journal.pone.0222807)
Supplement: S1 Text — Text of the email sent to all past Community for Data Integration project principal investigators to ask for information about their project outputs. (PDF) [file pone.0222807.s004.pdf]

S1 Text. Email to Principal Investigators.

Text of the email sent to all past Community for Data Integration project principal investigators to ask for information about their project outputs.

Dear (Project contact),

I am doing a study on the status and sustainability of past CDI funded projects, for presentation in mid-July. Could you please help me with the (year) project “(title)”?

1. Are you the best point of contact for this project, if not, who is?
2. Do you consider the outputs of the project still operational/accessible?
3. Have you sought additional funds outside of CDI to continue or sustain this project? (If so, where, and were you successful?)
4. Please share any comments about specific users of your project outcomes, what you consider the impact of your project (e.g. knowledge transfer, network building, scientific research, URLs), and issues of sustainability.

If you could please answer at your earliest convenience, or by July 7, it would be extremely helpful to me. Even very short answers will be useful.

Thank you for your time!

Sincerely,
Leslie Hsu
CDI Coordinator

Supporting information for Hsu, Hutchison, and Langseth, Measuring sustainability of seed-funded Earth science informatics projects.
